# Jejunogastric Intussusception: A Rare Complication of Gastric Surgery

**DOI:** 10.1155/2013/838360

**Published:** 2013-06-13

**Authors:** Gokhan Cipe, Fatma Umit Malya, Mustafa Hasbahceci, Yeliz Emine Ersoy, Oguzhan Karatepe, Mahmut Muslumanoglu

**Affiliations:** Bezmialem Vakıf University, Department of General Surgery, 34093 Istanbul, Turkey

## Abstract

Jejunogastric intussusception is a rare complication of gastric surgery. It usually presents with severe epigastric pain, vomiting, and hematemesis. A history of gastric surgery can help in making an accurate and early diagnosis which calls forth an urgent surgical intervention. Only reduction or resection with revision of the previously performed anastomosis is the choice which is decided according to the operative findings. We present a case of JGI in a patient with a history of Billroth II operation diagnosed by computed tomography. At emergent laparotomy, an efferent loop type JGI was found. Due to necrosis, resection of the intussuscepted bowel with Roux-en-Y anastomosis was performed. Postoperative recovery was uneventful.

## 1. Introduction

Jejunogastric intussusception (JGI) is a rare complication of gastrectomy with an incidence of 0.1% [1]. It is thought that it can occur any time after several types of the gastric operations including gastrojejunostomy and Billroth II resection [2–4]. A mortality rate of 10% and even as high as of 50% has been reported if surgical intervention has been delayed [5, 6], therefore, early diagnosis of this condition is mandatory. Although a history of gastric surgery may help in making such a diagnosis, preoperative awareness of this condition has been reported to be difficult in most of the cases.

In this paper, we aim to report a case of JGI with regard to its presentation, diagnosis, and surgical treatment.

## 2. Case Report

**Figure 1:**
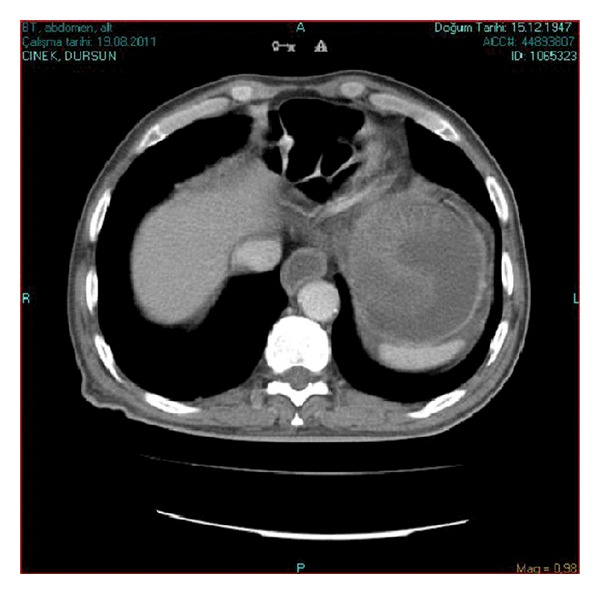
Emergency CT scan of the abdomen. Dilated stomach with intragastric nonhomogeneous mass compatible with bowel loops.

**Figure 2:**
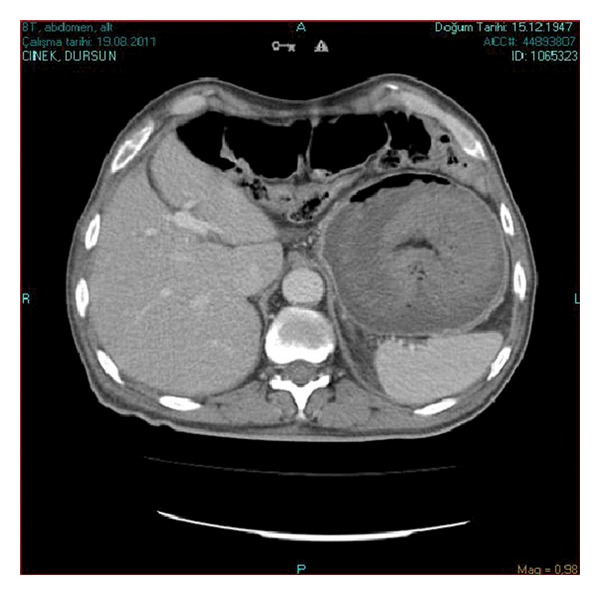
Emergency CT scan of the abdomen. Another section showing a dilated stomach with intragastric nonhomogeneous mass compatible with bowel loops.

**Figure 3:**
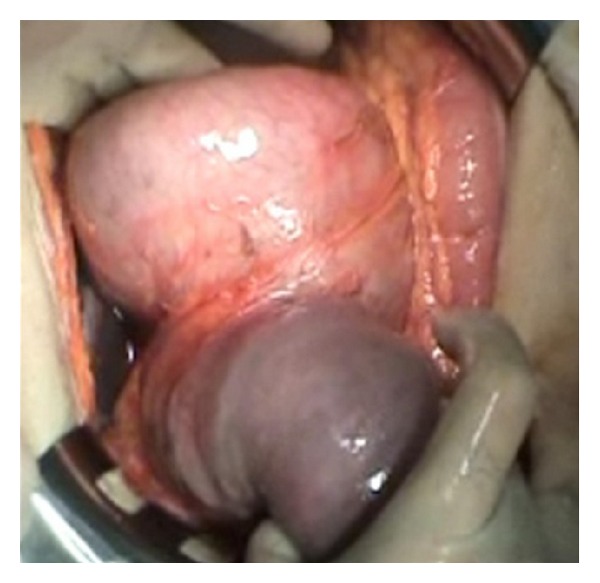
Invagination of the efferent loop to the stomach.

**Figure 4:**
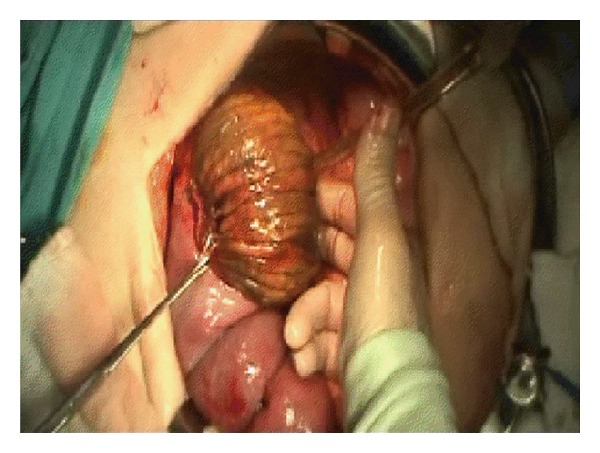
Another view of invagination of the efferent loop to the stomach.

A 63-year-old male patient was admitted to the hospital with severe colicky epigastric pain followed by hematemesis. There was a past history of gastric surgery (Billroth II operation), which had been performed 23 years previously for peptic ulcer disease. On physical examination, there was a mildly distended abdomen, epigastric tenderness, and a vague feeling of an epigastric mass on deep palpation. The usual laboratory investigation was unremarkable. A computed tomography showed a distended stomach containing a nonhomogeneous mass (Figure 1). The diagnosis of JGI was established, and an emergent laparotomy was performed. At laparotomy, an ischemic efferent loop was found to be intussuscepted in a retrograde manner into the gastric lumen (Figures 2 and 3). Following reduction of this jejunal segment, resection with Roux-en-Y anastomosis was performed due to ongoing necrosis (Figure 4). The patient was discharged at the fifth postoperative day without any complaint.

## 3. Discussion

The term retrograde intussusception (invagination) was first introduced by John Hunter to define an invagination of the intussusceptum in an antiperistaltic or proximal direction as opposed to the usual peristaltic or distal direction [7]. Intussusception is an uncommon condition that may arise at any age. It is usually seen in childhood, and only 5% of cases occur in adults [8]. Jejunogastric intussusception is a rare complication of gastrojejunostomy, Billroth II gastrectomy, and Roux-en-Y anastomosis. There were less than 200 published cases since its first description in 1914 by Bozzi in a patient with gastrojejunostomy [3]. In 1922, Lundberg reported a case of JGI in a patient with a history of Billroth II resection [4]. According to the type of intussuscepted loop, JGI is classified into three types: type I, antegrade or afferent loop intussusception; type II, retrograde or efferent loop intussusception; and type III, combined form [5]. Efferent loop JGI is seen in 80% of the cases as in the present case, while others account for the remaining 20% [5]. The exact mechanism of JGI is still not well understood. Long afferent loop, jejunal spasm with abnormal motility, increased mobility of the efferent loop, and adhesions leading to the intussusception of a more mobile segment into a fixed segment may be the underlying causes [1]. It is also postulated that increased intra-abdominal pressure, a dilated atonic stomach especially after vagotomy, and retrograde peristalsis may be responsible for the development of JGI [1]. 

Two different forms of JGI have been described according to its clinical presentation [9]. In the acute form, incarceration and strangulation of the intussuscepted loop causing acute severe epigastric pain, vomiting, and subsequently, hematemesis generally occur. However, spontaneous reduction is usual in the chronic type. A palpable abdominal mass can be observed in almost half of the cases [1, 10]. It should be kept in mind that a sudden onset of epigastric pain, vomiting and subsequent hematemesis, and a palpable epigastric mass in a patient with a previous gastric surgery can be important diagnostic clues for JGI [1]. Therefore, carefully taken history with good physical examination helps to suspect this rare condition in a gastrectomized patient as in this case.

Early diagnosis of the acute form is of paramount importance. The first specific diagnostic study should be an emergent endoscopy which is carried out by endoscopists aware of JGI and its endoscopic picture. Computed tomography allows the differentiation of the distinct stages of the disease. It shows a dilated stomach with intragastric filling by the bowel loops. In spite of the endoscopic and imaging findings, most reported cases of JGI were diagnosed at surgery [9]. 

Surgical options include only reduction or resection of the compromised bowel with revision of the anastomosis depending on the conditions found during the operation [6]. It could not be possible to prevent necrosis in the intussuscepted jejunal bowel despite early diagnosis and immediate surgical intervention. Therefore, resection with revision of the previous anastomosis can be the most appropriate surgical method in a patient with acute JGI.

## 4. Conclusions

JGI is a rare life-threatening complication of gastric surgery which is often diagnosed at surgery. Although endoscopy and computed tomography may be helpful during preoperative evaluation, laparotomy is usually required for the correct diagnosis. Early surgical intervention is the most important factor for prevention of morbidity and mortality.
